# Supplementary material for: Social media strategies used to translate knowledge and disseminate clinical neuroscience information to healthcare users: A systematic review
Source: PLOS Digit Health. 2025 Apr 8;4(4):e0000778. doi: 10.1371/journal.pdig.0000778 (PMC11978067; doi:10.1371/journal.pdig.0000778)
Supplement: S1 File — (DOCX) [file pdig.0000778.s001.docx]

**
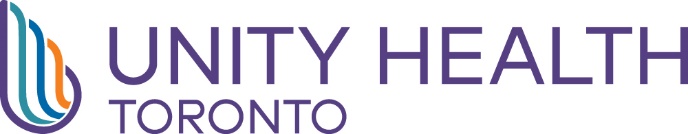
**

Requestor(s) name and email:

[rbaran2@uwo.ca](mailto:rbaran2@uwo.ca)

[Melissa.Carpino@unityhealth.to](mailto:Melissa.Carpino@unityhealth.to)

[CusimanoM@smh.ca](mailto:CusimanoM@smh.ca)

**Date:** 16 July 2021

**Information Specialist**:

David Lightfoot

Information Specialist

Health Sciences Library

30 Bond Street, Toronto, Ontario M5B 1W8

416.864.6060 X77546

david.lightfoot@unityhealth.to

Table of Contents

[Research Question: What roles do social media play in education practitioners and patients about neurological diseases and conditions? 2](#_Toc77607260)

[Description 2](#_Toc77607261)

[Number of results per database 2](#_Toc77607262)

[Search Strategies 3](#_Toc77607263)

[All Ovid Medline <1946 - present> 3](#_Toc77607264)

[CINAHL 4](#_Toc77607265)

[EBM Reviews - Cochrane Database of Systematic Reviews <2005 to July 14, 2021> 6](#_Toc77607266)

[EBM Reviews - ACP Journal Club <1991 to June 2021> 6](#_Toc77607267)

[EBM Reviews - Database of Abstracts of Reviews of Effects <1st Quarter 2016> 6](#_Toc77607268)

[EBM Reviews - Cochrane Clinical Answers <June 2021> 6](#_Toc77607269)

[EBM Reviews - Cochrane Central Register of Controlled Trials <June 2021> 6](#_Toc77607270)

[EBM Reviews - Cochrane Methodology Register <3rd Quarter 2012> 6](#_Toc77607271)

[EBM Reviews - Health Technology Assessment <4th Quarter 2016> 6](#_Toc77607272)

[EBM Reviews - NHS Economic Evaluation Database <1st Quarter 2016> 6](#_Toc77607273)

[Database 3 [use heading 2] 7](#_Toc77607274)

[Scopus 8](#_Toc77607275)

[Results: Titles & Abstracts [use heading 1] 9](#_Toc77607276)

# Research Question: What social media strategies are used to translate knowledge and disseminate clinical neuroscience research to healthcare users (including patients, HCPs, and the general public)? How effective are these strategies at disseminating clinical neuroscience research to the aforementioned stakeholders?

## Description

Medline, Embase, and the Cochrane Database of Systematic Reviews were all searched using indexed words and keywords appearing in the title, abstract, and author supplied keywords. Common acronyms were searched in abstracts only.

# Number of results per database

| **Databases Searched** | **Date of Search** | **Number of Results** |
| --- | --- | --- |
| All Ovid Medline <1946 - present> | 16 July 2021 | 480 |
| CINAHL | 16 July 2021 | 626 |
| Cochrane Databases | 16 July 2021 | 95 |
| Embase | 16 July 2021 | 2592 |
| Scopus | 16 July 2021 | 74 |
| Web of Science | 16 July 2021 | 676 |

TOTAL SUBMITTED FOR REVIEW: 4543

| TOTAL SUBMITTED FOR REVIEW | 4543 |
| --- | --- |
| Deduplicated: approx | 3702 (check against number in COVIDENCE |

#

# Search Strategies

## All Ovid Medline <1946 - present>

1 exp Neurosciences/ 22672

2 neuroscience.mp. or Neurosciences/ 37832

3 exp neurology/ or *neuropathology/ or *neurotology/ 19284

4 exp *nervous system diseases/ or *"autoimmune diseases of the nervous system"/ or *autonomic nervous system diseases/ or *central nervous system diseases/ or *chronobiology disorders/ or *cranial nerve diseases/ or *demyelinating diseases/ or *nervous system malformations/ or *nervous system neoplasms/ or *neurocutaneous syndromes/ or *neurodegenerative diseases/ or *neurologic manifestations/ or *neuromuscular diseases/ or *neurotoxicity syndromes/ or *restless legs syndrome/ or *sleep wake disorders/ or *trauma, nervous system/ 2234061

5 exp central nervous system diseases/ or brain diseases/ or exp brain injuries/ 1502829

6 aneurysm:.ti,ab,kf. 130091

7 brain disease*.ti,ab,kf. 11333

8 (brain injur* adj2 traumatic).ti,ab,kf. 41447

9 brain neoplas:.ti,ab,kf. 6197

10 ((Brain adj2 tumor:) or (Brain adj2 tumour:)).ti,ab,kf. 48872

11 cerebrovascular.ti,ab,kf. 62049

12 Concussion:.ti,ab,kf. 10243

13 concussive.ti,ab,kf. 1451

14 concussive.ti,ab,kf. 1451

15 dementia.tw. 115688

16 hematoma:.ti,ab,kf. 49655

17 stroke.ti,ab,kf. 265569

18 1 or 2 or 3 or 4 or 5 or 6 or 7 or 8 or 9 or 10 or 11 or 12 or 13 or 14 or 15 or 16 or 17 2771935

19 exp Social Media/ or "Text Messaging"/ 14093

20 (tweets or tweeting or blog or blogging or blogger or texting or "text messag*" or "social media" or "online social network*" or "Baidu Tieba" or Douban or Facebook or Foursquare or "Google app*" or Influenster or Instagram or Kuaishou or Lasso or Linkedin or Messenger or Meetup or Mocospace or Snapchat or "snap chat" or Pinterest or Qzone or "Sina Weibo" or Skype or Steemit or "Tencent QQ" or Tik Tok or Tiktok or Tinder or Tumblr or Twitter or YouTube or "You Tube" or Reddit or Vero or Viber or SMS or VKontakte or Wattpad or Wechat or WhatsApp or Xanga or XING).ti. 27930

21 ("SMS 201 995" or "Snyder Robinson Syndrome").mp. [mp=title, abstract, original title, name of substance word, subject heading word, floating sub-heading word, keyword heading word, organism supplementary concept word, protocol supplementary concept word, rare disease supplementary concept word, unique identifier, synonyms] 797

22 (messenger adj3 RNA).mp. 393556

23 21 or 22 394333

24 20 not 23 17814

25 vlog:.tw. 67

26 24 or 25 17866

27 18 and 26 539

28 exp animals/ not humans.sh. 4861567

29 27 not 28 480

30 from 29 keep 1-480 480

## CINAHL

626

| S1 | (MM "Neurosciences+") OR (MM "Neuroanatomy") OR (MM "Neurobiology") OR (MM "Neurophysiology") |  |  |  |
| --- | --- | --- | --- | --- |
| S2 | (MM "Neurology") |  |  |  |
| S3 | (MM "Neurodegenerative Diseases") OR (MH "Nervous System Diseases+") OR (MM "Autoimmune Diseases of the Nervous System") OR (MM "Autonomic Nervous System Diseases") OR (MM "Central Nervous System Diseases") OR (MM "Chronobiology Disorders") OR (MM "Cranial Nerve Diseases") OR (MM "Demyelinating Diseases") OR (MM "Nervous System Neoplasms") OR (MM "Neurocutaneous Syndromes") OR (MM "Alzheimer's Disease") OR (MM "Chronic Traumatic Encephalopathy") OR (MM "Heredodegenerative Disorders, Nervous System") OR (MM "Motor Neuron Diseases") OR (MM "Multiple System Atrophy") OR (MM "Paraneoplastic Syndromes, Nervous System") OR (MM "Parkinsonian Disorders") OR (MM "Postpoliomyelitis Syndrome") OR (MM "Prion Diseases") OR (MM "Shy-Drager Syndrome") OR (MM "Supranuclear Palsy, Progressive") OR (MM "Synucleinopathies") OR (MM "TDP-43 Proteinopathies") OR (MM "Neurologic Manifestations") OR (MM "Neuromuscular Diseases") OR (MM "Neurotoxicity Syndromes") |  |  |  |
| S4 | (MH "Aneurysm+") OR "aneurysm" |  |  |  |
| S5 | (MM "Brain Diseases") OR (MM "Acute Febrile Encephalopathy") OR (MM "Amblyopia") OR (MM "Auditory Diseases, Central") OR (MM "Basal Ganglia Diseases") OR (MM "Brain Abscess") OR (MM "Brain Damage, Chronic") OR (MM "Brain Diseases, Metabolic") OR (MM "Brain Injuries") OR (MM "Brain Neoplasms") OR (MM "Cerebellar Diseases") OR (MM "Cerebral Edema") OR (MM "Cerebrovascular Disorders") OR (MM "Dyke-Davidoff-Masson Syndrome") OR (MM "Dementia") OR (MM "Encephalitis") OR (MM "Epilepsy") OR (MM "Headache") OR (MM "Hydrocephalus") OR (MM "Hypothalamic Diseases") OR (MM "Hypoxia, Brain") OR (MM "Intracranial Hypertension") OR (MM "Central Nervous System Infections") OR (MM "Toxoplasmosis, Cerebral") OR (MM "Thalamic Diseases") OR (MM "Reye's Syndrome") OR (MM "Hyperekplexia") OR (MM "Movement Disorders") OR (MM "Ocular Motility Disorders") OR (MM "Pneumocephalus") OR (MM "Spinal Cord Diseases") |  |  |  |
| S6 | (MM "Brain Neoplasms+") OR (MM "Diffuse Intrinsic Pontine Glioma") OR (MM "Neoplasms, Cystic, Mucinous, and Serous") OR (MM "Neoplasms, Glandular and Epithelial") OR (MM "Neuroectodermal Tumors, Primitive") OR (MM "Neoplasms, Complex and Mixed") OR (MM "Neoplasm Regression, Spontaneous") OR (MM "Neoplasms, Connective and Soft Tissue") OR (MM "Neoplasms, Nerve Tissue") OR (MM "Infratentorial Neoplasms") OR (MM "Hypothalamic Neoplasms") OR (MM "Neoplasms, Germ Cell and Embryonal") OR "Brain tumor" |  |  |  |
| S7 | (MH "Brain Concussion+") OR (MM "Postconcussion Syndrome") OR (MM "Commotio Cordis") OR (MM "Brain Contusions") OR "concussion" |  |  |  |
| S8 | (MM "Hematoma+") OR "hematoma" OR (MM "Hematoma, Subdural, Chronic") OR (MM "Hematoma, Subdural, Acute") OR (MM "Hematoma, Subdural") OR (MM "Hematoma, Epidural") OR (MM "Basal Ganglia Hemorrhage") |  |  |  |
| S9 | (MH "Cerebrovascular Disorders+") OR "cerebrovascular" |  |  |  |
| S10 | S1 OR S2 OR S3 OR S4 OR S5 OR S6 OR S7 OR S8 OR S9 |  |  |  |
| S11 | S1 OR S2 OR S3 OR S4 OR S5 OR S6 OR S7 OR S8 OR S9 OR S10 |  |  |  |
| S12 | (MH "Social Media+") OR "social media" OR (MM "Communications Media") OR (MM "Social Worker Attitudes") OR (MM "Social Workers") OR (MM "Social Behavior") OR (MM "Social Work") OR (MM "Social Adjustment") OR (MM "Social Anxiety Disorders") OR (MM "Students, Social Work") OR (MM "Social Work, Psychiatric") |  |  |  |
| S13 | (MH "Text Messaging+") OR "text messaging" OR (MM "Instant Messaging") |  |  |  |
| S14 | TI tweets or tweeting or blog or blogging or blogger or texting or "text messag*" or "social media" or "online social network*" or "Baidu Tieba" or Douban or Facebook or Foursquare or "Google app*" or Influenster or Instagram or Kuaishou or Lasso or Linkedin or Messenger or Meetup or Mocospace or Snapchat or "snap chat" or Pinterest or Qzone or "Sina Weibo" or Skype or Steemit or "Tencent QQ" or Tik Tok or Tiktok or Tinder or Tumblr or Twitter or YouTube or "You Tube" or Reddit or Vero or Viber or SMS or VKontakte or Wattpad or Wechat or WhatsApp or Xanga or XING) |  |  |  |
| S15 | TI vlog OR TX vlogging OR TX Vlogger |  |  |  |
| S16 | S12 OR S13 OR S14 OR S15 |  |  |  |
| S17 | S11 AND S16 |  |  |  |
| S18 | (MH "Patients+") OR "Patients" OR (MM "Health Personnel as Patients") OR (MM "Stroke Patients") OR (MM "Rehabilitation Patients") OR (MM "Terminally Ill Patients") |  |  |  |
| S19 | S17 AND S18 |  |  |  |

Bottom of Form

##

## EBM Reviews – All Cochrane Reviews <2005 to July 14, 2021>

1 exp Neurosciences/ 74

2 neuroscience.mp. or Neurosciences/ 1021

3 exp neurology/ or *neuropathology/ or *neurotology/ 68

4 exp *nervous system diseases/ or *"autoimmune diseases of the nervous system"/ or *autonomic nervous system diseases/ or *central nervous system diseases/ or *chronobiology disorders/ or *cranial nerve diseases/ or *demyelinating diseases/ or *nervous system malformations/ or *nervous system neoplasms/ or *neurocutaneous syndromes/ or *neurodegenerative diseases/ or *neurologic manifestations/ or *neuromuscular diseases/ or *neurotoxicity syndromes/ or *restless legs syndrome/ or *sleep wake disorders/ or *trauma, nervous system/ 62327

5 exp central nervous system diseases/ or brain diseases/ or exp brain injuries/ 48597

6 aneurysm:.ti,ab,kf. 5064

7 brain disease*.ti,ab,kf. 274

8 (brain injur* adj2 traumatic).ti,ab,kf. 4216

9 brain neoplas:.ti,ab,kf. 29

10 ((Brain adj2 tumor:) or (Brain adj2 tumour:)).ti,ab,kf. 1974

11 cerebrovascular.ti,ab,kf. 6354

12 Concussion:.ti,ab,kf. 686

13 concussive.ti,ab,kf. 180

14 concussive.ti,ab,kf. 180

15 dementia.tw. 14957

16 hematoma:.ti,ab,kf. 3997

17 stroke.ti,ab,kf. 59234

18 1 or 2 or 3 or 4 or 5 or 6 or 7 or 8 or 9 or 10 or 11 or 12 or 13 or 14 or 15 or 16 or 17 154403

19 exp Social Media/ or "Text Messaging"/ 1146

20 (tweets or tweeting or blog or blogging or blogger or texting or "text messag*" or "social media" or "online social network*" or "Baidu Tieba" or Douban or Facebook or Foursquare or "Google app*" or Influenster or Instagram or Kuaishou or Lasso or Linkedin or Messenger or Meetup or Mocospace or Snapchat or "snap chat" or Pinterest or Qzone or "Sina Weibo" or Skype or Steemit or "Tencent QQ" or Tik Tok or Tiktok or Tinder or Tumblr or Twitter or YouTube or "You Tube" or Reddit or Vero or Viber or SMS or VKontakte or Wattpad or Wechat or WhatsApp or Xanga or XING).ti. 2928

21 ("SMS 201 995" or "Snyder Robinson Syndrome").mp. [mp=ti, ab, tx, kw, ct, ot, sh, hw] 98

22 (messenger adj3 RNA).mp. 2781

23 21 or 22 2879

24 20 not 23 2795

25 vlog:.tw. 10

26 24 or 25 2804

27 18 and 26 95

## Embase Classic+Embase <1947 to 2021 July 16>

1 exp neuroscience/ or neuroscience.mp. 277554

2 neurology/ or exp neurology/ 69404

3 neuropathology.mp. or exp neuropathology/ 64895

4 nervous system diseases.mp. or exp neurologic disease/ 4025349

5 brain disease/ or exp central nervous system disease/ 2924276

6 aneurysm.mp. or exp aneurysm/ 217465

7 (brain adj2 injur*).ti,ab,kw. 111140

8 exp cerebrovascular disease/ or cerebrovascular.mp. 746016

9 concussion*.ti,ab,kw. 14606

10 concussive:.ti,ab,kw. 2280

11 dementia:.ti,ab,kw. 182736

12 hematoma:.ti,ab,kw. 72464

13 stroke:.ti,ab,kw. 444994

14 1 or 2 or 3 or 4 or 5 or 6 or 7 or 8 or 9 or 10 or 11 or 12 or 13 4554953

15 social media.mp. or exp social media/ 32387

16 texting.mp. or exp text messaging/ 6289

17 (tweets or tweeting or blog or blogging or blogger or texting or "text messag*" or "social media" or "online social network*" or "Baidu Tieba" or Douban or Facebook or Foursquare or "Google app*" or Influenster or Instagram or Kuaishou or Lasso or Linkedin or Messenger or Meetup or Mocospace or Snapchat or "snap chat" or Pinterest or Qzone or "Sina Weibo" or Skype or Steemit or "Tencent QQ" or Tik Tok or Tiktok or Tinder or Tumblr or Twitter or YouTube or "You Tube" or Reddit or Vero or Viber or SMS or VKontakte or Wattpad or Wechat or WhatsApp or Xanga or XING).ti. 31879

18 vlog:.tw. 53

19 or/15-18 57499

20 ("SMS 201 995" or "Snyder Robinson Syndrome").mp. 1208

21 (messenger adj3 RNA).mp. 619537

22 20 or 21 620714

23 19 not 22 46708

24 14 and 23 2857

25 limit 24 to human 2592

Scopus 74

( TITLE-ABS-KEY ( neuroscience*  OR  neurology  OR  neurologic  OR  cerebrovascular  OR  aneurysm  OR  brain  AND disease  OR  brain  AND injury  OR  concussion  OR  concussive  OR  neurophys:  OR  hematoma  OR  stroke )  AND  TITLE-ABS-KEY ( tweets  OR  tweeting  OR  blog  OR  blogging  OR  blogger  OR  texting  OR  "text messag*"  OR  "social media"  OR  "online social network*"  OR  "Baidu Tieba"  OR  douban  OR  facebook  OR  foursquare  OR  "Google app*"  OR  influenster  OR  instagram  OR  kuaishou  OR  lasso  OR  linkedin  OR  messenger  OR  meetup  OR  mocospace  OR  snapchat  OR  "snap chat"  OR  pinterest  OR  qzone  OR  "Sina Weibo"  OR  skype  OR  steemit  OR  "Tencent QQ"  OR  tik  AND tok  OR  tiktok  OR  tinder  OR  tumblr  OR  twitter  OR  youtube  OR  "You Tube"  OR  reddit  OR  vero  OR  viber  OR  sms  OR  vkontakte  OR  wattpad  OR  wechat  OR  whatsapp  OR  xanga  OR  xing  OR  vlog  OR  vlogger ) ) 

Web of Science 676

( TITLE-ABS-KEY ( neuroscience*  OR  neurology  OR  neurologic  OR  cerebrovascular  OR  aneurysm  OR  brain  AND disease  OR  brain  AND injury  OR  concussion  OR  concussive  OR  neurophys:  OR  hematoma  OR  stroke )  AND  TITLE-ABS-KEY ( tweets  OR  tweeting  OR  blog  OR  blogging  OR  blogger  OR  texting  OR  "text messag*"  OR  "social media"  OR  "online social network*"  OR  "Baidu Tieba"  OR  douban  OR  facebook  OR  foursquare  OR  "Google app*"  OR  influenster  OR  instagram  OR  kuaishou  OR  lasso  OR  linkedin  OR  messenger  OR  meetup  OR  mocospace  OR  snapchat  OR  "snap chat"  OR  pinterest  OR  qzone  OR  "Sina Weibo"  OR  skype  OR  steemit  OR  "Tencent QQ"  OR  tik  AND tok  OR  tiktok  OR  tinder  OR  tumblr  OR  twitter  OR  youtube  OR  "You Tube"  OR  reddit  OR  vero  OR  viber  OR  sms  OR  vkontakte  OR  wattpad  OR  wechat  OR  whatsapp  OR  xanga  OR  xing  OR  vlog  OR  vlogger ) )
