# Supplementary material for: Social media strategies used to translate knowledge and disseminate clinical neuroscience information to healthcare users: A systematic review
Source: PLOS Digit Health. 2025 Apr 8;4(4):e0000778. doi: 10.1371/journal.pdig.0000778 (PMC11978067; doi:10.1371/journal.pdig.0000778)
Supplement: S1 Table — (PDF) [file pdig.0000778.s003.pdf]

S1 Table. List of studies excluded at full-text screening with brief reasoning (N = 17).

First Author	Year of Publication	Article Title	Reason for Exclusion
Abcejo, AS	2021	Twitter Journal Clubs in Perioperative Neuroscience: Opportunities and Challenges	Editorial, not a peer-reviewed original article.
Ahmed, O	2016	A picture tells a thousand words: A content analysis of concussion-related images online	Not an outcome of interest. Only examined the accuracy of concussion-related images and content disseminated on social media but did not measure its efficacy pertaining to knowledge translation.
Akre, C	2018	Children's at Home: Pilot Study Assessing Dedicated Social Media for Parents of Adolescents with Neurofibromatosis Type 1	Not an intervention or outcome of interest. Only examined parents sharing their non-clinical personal experiences on social media and only measured the feasibility and acceptability of the program.
Akre, C	2016	Children's at home: Dedicated social media for parents of adolescents with neurofibromatosis type 1	Conference abstract only, not a full-text article.
Alotaibi, NM	2017	The Use of Social Media Communications in Brain Aneurysms and Subarachnoid Hemorrhage: A Mixed-Method Analysis	Lack of an intervention. Only conducted a thematic analysis of pre-existing social media communications.
Asano, E	2007	A public outreach in epilepsy surgery using a serial novel on BLOG: a preliminary report	Short communication, not a full-text peer-reviewed original article.
Bove, R	2019	Strengthening knowledge when data are scarce: The role of social media after AFM	Supplement only, not a full-text article.
Chan, TM	2014	Global emergency medicine journal club: Social media responses to the January 2014 online emergency medicine journal club on subarachnoid hemorrhage	Not an outcome of interest. Only evaluated the content of online discussions but did not measure any outcomes of knowledge translation.
Driver, S	2017	The social network-using social media to support individuals with TBI in a health promotion program	Abstract only, not a full-text article.
Foley, F	2019	Creating greater public understanding of dementia: Findings on the impact of a coalition-led national awareness programme	Poster presentation, not a full-text peer-reviewed original article.

Getchius, T	2015	Do social media improve dissemination of Clinical Practice Guidelines (CPG)?	Supplement only, not a full-text article.
Inge, KJ	2017	Evaluating the effectiveness of Facebook to impact the knowledge of evidence-based employment practices by individuals with traumatic brain injury: A knowledge translation random control study	Not an intervention of interest. Only disseminated information about employment practices but did not disseminate clinical neuroscience information.
Li, J	2024	Application of Hospital-Community-Family "Trinity" Remote Rehabilitation Guidance Based on Internet + WeChat Platform in Patients with Stroke After Discharge	Not an outcome of interest. Limb function recovery, activities of daily living, compliance score to rehabilitation nursing, and satisfaction survey score are not relevant outcomes of knowledge translation.
Peries, R	2016	Acceptance of social media to raise awareness on stroke in a local setting	Supplement only, not a full-text article.
Tan, C	2022	Effect of Continuous Nursing Model Based on WeChat Public Health Education on Self-Management Level and Treatment Compliance of Stroke Patients	Not an outcome of interest. Self-management level, quality of life, and treatment compliance are not relevant outcomes of knowledge translation.
Van Asbroeck, S	2021	Increasing knowledge on dementia risk reduction in the general population: Results of a public awareness campaign	The intervention included both social media and other non-social media methods (e.g., newspapers, leaflets). Therefore, we cannot determine whether the results are due to social media alone.
Wang, F	2017	Evaluation of a WeChat-based dementia-specific training program for nurses in primary care settings: A randomized controlled trial	The intervention involved both lectures and social media to disseminate dementia training. Therefore, we cannot determine whether the results are due to social media alone.
