# Supplementary figures and images for: Social media strategies used to translate knowledge and disseminate clinical neuroscience information to healthcare users: A systematic review
Source: PLOS Digit Health. 2025 Apr 8;4(4):e0000778. doi: 10.1371/journal.pdig.0000778 (PMC11978067; doi:10.1371/journal.pdig.0000778)

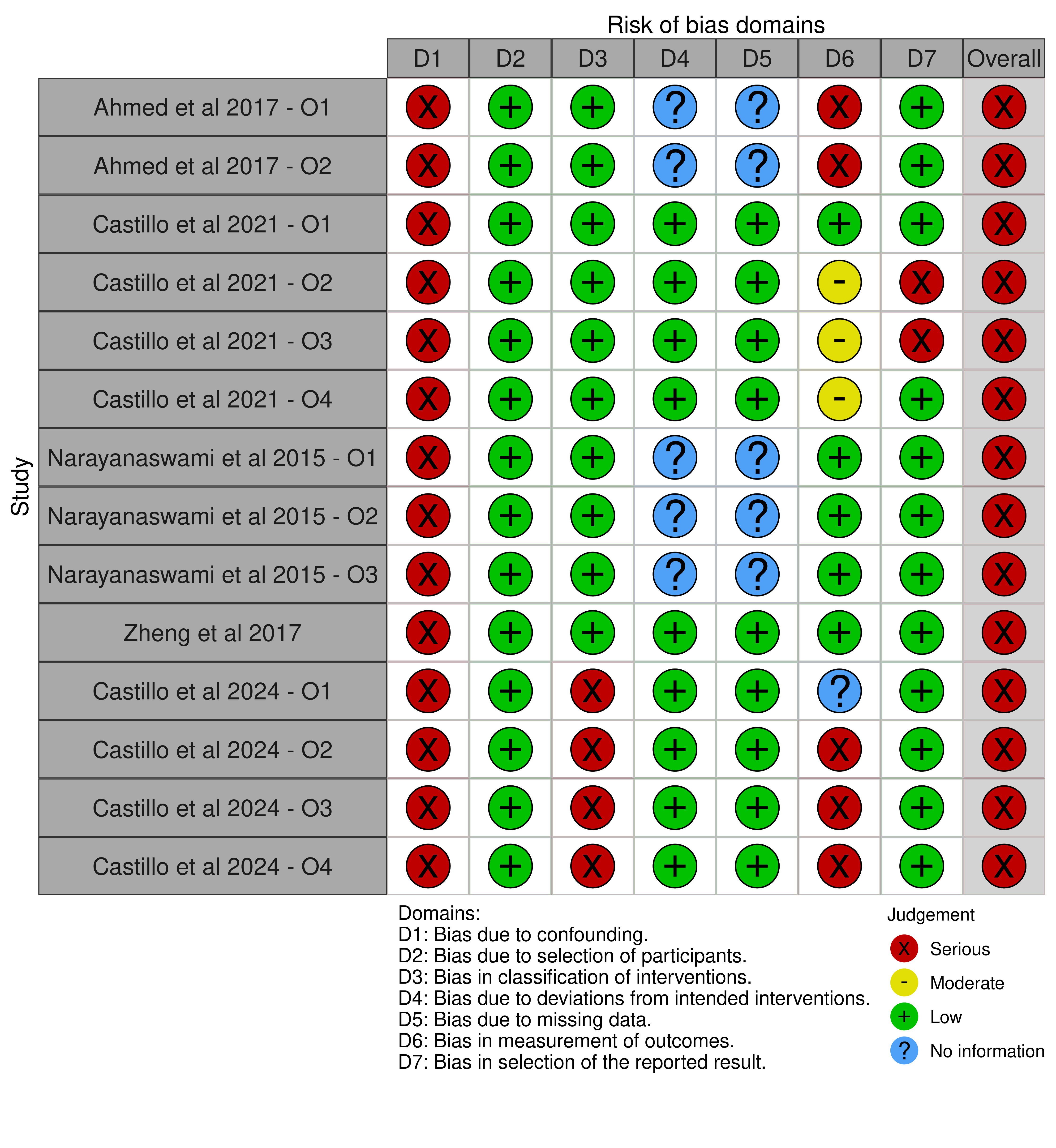

Supplement: S1 Fig — (TIF) [file pdig.0000778.s004.tiff]

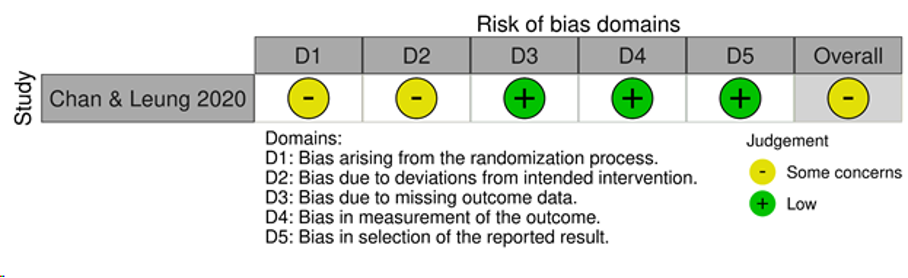

Supplement: S2 Fig — (TIF) [file pdig.0000778.s005.tiff]

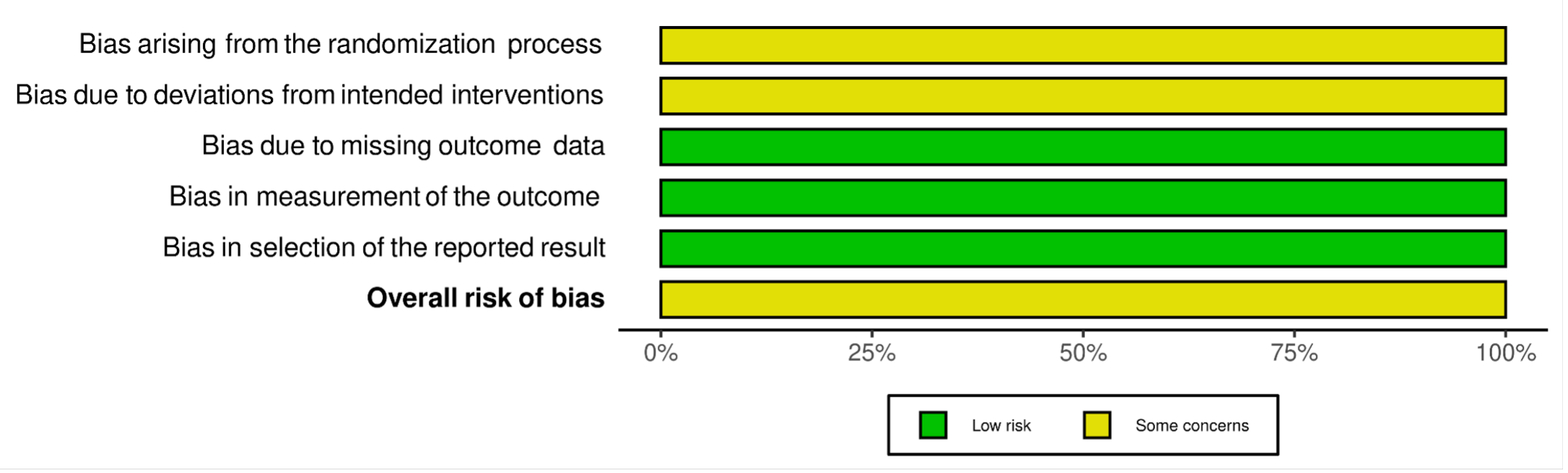

Supplement: S4 Fig — (TIF) [file pdig.0000778.s007.tiff]

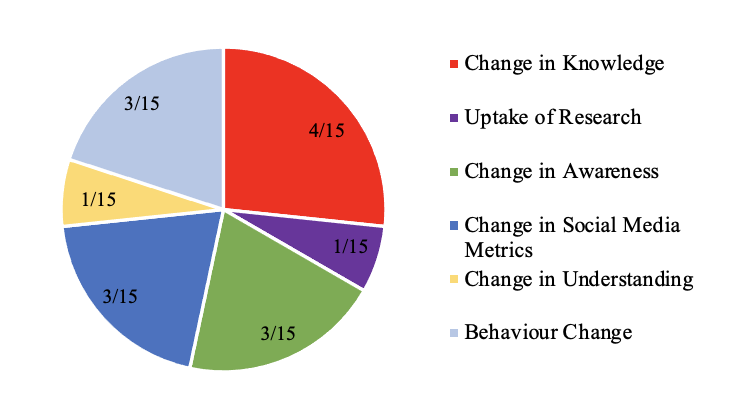

Supplement: S5 Fig — (TIF) [file pdig.0000778.s008.tiff]

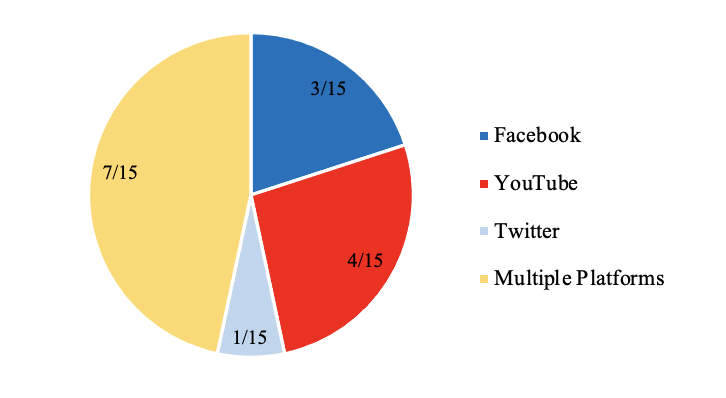

Supplement: S6 Fig — (TIF) [file pdig.0000778.s009.tiff]

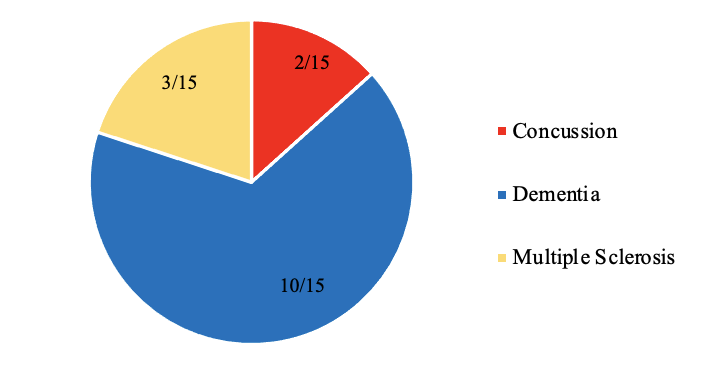

Supplement: S7 Fig — (TIF) [file pdig.0000778.s010.tiff]
